# An Intelligent Weather Station

**DOI:** 10.3390/s151229841

**Published:** 2015-12-10

**Authors:** Gonçalo Mestre, Antonio Ruano, Helder Duarte, Sergio Silva, Hamid Khosravani, Shabnam Pesteh, Pedro M. Ferreira, Ricardo Horta

**Affiliations:** 1 EasySensing—Intelligent Systems, Centro Empresarial de Gambelas, Pav A5, Campus de Gambelas, University of Algarve, 8005-139 Faro, Portugal; goncalomestre@easysensing.pt (G.M.); sergiosilva@easysensing.pt (S.S.); 2 Faculty of Science and Technology, Campus de Gambelas, University of Algarve, 8005-139 Faro, Portugal; hsduarte@ualg.pt (H.D.); hkhosravani@csi.fct.ualg.pt (H.K.); spesteh@csi.fct.ualg.pt (S.P.); 3 Centre for Intelligent Systems, IDMEC, Instituto Superior Técnico, 1049-001 Lisboa, Portugal; 4 LaSIGE, Faculdade de Ciências, Universidade de Lisboa, Portugal; pmf@ciencias.ulisboa.pt; 5 Rolear SA, 8001-906 Faro, Portugal; ricardohorta@rolear.pt

**Keywords:** weather station, self-powered device, neural networks, prediction, wireless communications, MOGA design

## Abstract

Accurate measurements of global solar radiation, atmospheric temperature and relative humidity, as well as the availability of the predictions of their evolution over time, are important for different areas of applications, such as agriculture, renewable energy and energy management, or thermal comfort in buildings. For this reason, an intelligent, light-weight, self-powered and portable sensor was developed, using a nearest-neighbors (NEN) algorithm and artificial neural network (ANN) models as the time-series predictor mechanisms. The hardware and software design of the implemented prototype are described, as well as the forecasting performance related to the three atmospheric variables, using both approaches, over a prediction horizon of 48-steps-ahead.

## 1. Introduction

There are a number of different weather stations available in the market. They provide measurements of atmospheric parameters such as solar radiation, air temperature and relative humidity, wind velocity and direction, atmospheric pressure and rainfall, the first three variables being the most common.

The atmospheric variables are measured by sensors that usually are connected to a data logger. These are powered by an external power supply, internal or external depending on the data logger. To avoid blackouts, some weather stations include a small size battery. One important point is that the conventional data loggers have a very limited storage size, and, in the case of a power shortage, they can lose some of their data. A conventional data logger is a passive device, meaning that they do not allow the addition of external routines and/or the reprogramming of the devices, with the objective of including control actions and prediction. Same data loggers are incorporated with programming options to change the acquisition parameters and the data availability.

With the evolution of technology, a few weather stations have a wireless interface integrated or offer it as an optional part. This wireless part typically uses the IEEE 802.11 or the IEEE 802.15.4 standards for communication. The ones that originally do not have the option to perform wireless data communication can be attached to a device that converts the data logger communication interface into a wireless communication, with the use of devices such as the NPort Z3150 (Moxa, Brea, CA, USA) [1] or the Waspmote (Libelium, Zaragoza, Spain) [2] that can be programmed to reach that goal.

A major field of current research consists in developing intelligent systems capable of integrating environmental data, to improve efficiency in the use of resources and to enable sustainable functioning of man-made utilities. This can be achieved by making an intelligent use of environmental variables forecasts [3], for applications such as photovoltaic plants and micro-grids [4,5,6], where global radiation and air temperature forecasts play an important role, in agricultural applications [7,8] for greenhouse environmental control and irrigation management, in energy management in buildings [9,10], where predictions of solar radiation, air temperature and relative humidity offer the possibility of important energy savings, as well as in sensor networks, with nodes powered by miniature solar harvesters, where forecasts of solar radiation reduces the risk of temporary depletion and increases their utility [11]. These selected works, which are a small subset of a much larger body of research, undoubtedly illustrate the need of a weather station that, besides measurement of atmospheric variables, provides their forecasts autonomously, and makes them available wirelessly to the application at hand.

**Figure 1:**
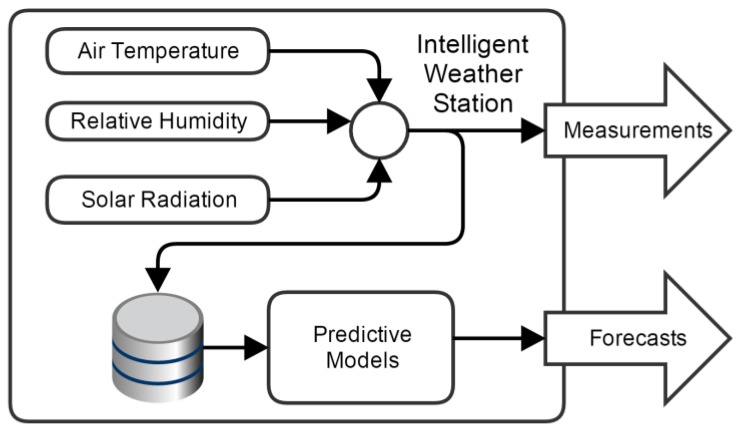
Simplified weather station signal-flow diagram.

The intelligent weather station described in this paper is a follow-up of the work described in [12]. It measures three atmospheric variables: Global solar radiation, air temperature and relative humidity. These measurements are carried at a user-specified time interval. In addition, it predicts the evolution of each variable in a prediction horizon up to 48 steps-ahead. The prediction step can coincide with the measurement sampling time, or it can be a multiple of it. In the latter case, the average value of each variable is computed, over the prediction step. In the case presented here, the sampling time is 1 min and the prediction step is 5 min, which means that the maximum prediction horizon is 4 h. The system was built with the objective of being self-sufficient, regarding electrical energy. In addition, to facilitate its deployment, it incorporates wireless communication based on the wireless IEEE 802.15.4 [13] standard. The weather station functional and signal flow diagram is presented in Figure 1.

This article is organized as follows. The hardware and software implementation are presented in Section 2 and Section 3, respectively. In Section 4, the working prototype is described and in Section 5 the prediction functionality is discussed. Results are shown in Section 6. Finally, conclusions are drawn in Section 7.

## 2. Hardware

The weather station development began with the search and selection of the hardware. As explained previously, it was necessary that a unit that could execute three predictive models in a multi-step fashion, store the data, enable the implementation of wireless communication and that provided an interface to connect other hardware, such as sensors. Besides the computational requirements, it was also necessary to take into account the device power consumption since the unit must be self-powered. In order to fulfill all requirements, the Raspberry PI B+ [14] was selected. It is a mini-computer created by the Raspberry foundation, which will be denoted in the sequel as RPI. To work with an RPI, an Operating System (OS) is compulsory. The operating system must be installed on a Secure Digital (SD), which must be permanently inserted on the RPI. There is a wide variety of OSs available for the RPI, most of them Linux based. The main differences between operating systems are their base distribution and goal; some are developed to fulfill specific purposes as a media center or a web server, where others are multi-purpose. From the available options, the Raspbian OS [15], an OS based on the Debian GNU/Linux Distribution [16], compiled and optimized for RPI, was selected. This Linux distribution was chosen due to its stability, support and the fact that it is multi-purpose.

With the weather station core selected, the development was shifted to the sensors employed to measure the atmospheric variables considered. The chosen sensors were the SHT75 [17] from Sensirion, to measure air temperature and relative humidity, and the SP-110 [18] from Apogee Instruments, to measure global solar radiation.

The SHT75 sensor is a 4 pin digital output sensor that uses a unique capacitive sensor to measure relative humidity and a band-gap sensor to measure air temperature. It was chosen mainly for its resolution, 0.05% for relative humidity and 0.01 °C for temperature. Another important factor was its stability over different atmospheric conditions. To obtain the most reliable data and to avoid problems with atmospheric conditions such as direct sun light exposure and rain, a solar radiation shield from Davis Instruments [19] was considered.

For the global solar radiation measurements, the SP-110 pyranometer was employed. The SP-110 is a self-powered sensor with a sensitivity of 0.20 mV per W/m^2^ and a resolution of 5 W/m^2^. The sensor has an analog output with a range from 0 mV to 250 mV. Since the RPI does not have analog ports, it was necessary to incorporate an analog to digital converter (ADC) to use this sensor. The chosen ADC uses an I2C interface and it has 12 bits of resolution [20].

Besides the sensors, a push button and a toggle button were also incorporated in the weather station design. The need for these buttons is due to the fact that the RPI does not have a power or reset button. Therefore a push button was added to work like a reset button: it makes changes to some configurations present in the RPI and restarts the device. The toggle button was added to provide power saving and debug modes on the weather station.

Once the main hardware was selected, a power consumption study was performed on the unit. During the study, it was found that, during normal use, it would consume around 300 mA without any devices attached to it. After disabling some unnecessary software services, mostly related to the Ethernet, USB and HDMI interfaces, the consumption dropped to around 150 mA. Based on that, it was decided to have two types of modes: A power saving mode, where those services were turned off, saving power but disabling debugging; and a debug mode, where all the services were on and debugging was possible. The programming related to these buttons will be discussed in the Software section.

As pointed out earlier, an important functionality of the device is its ability to communicate wirelessly using an IEEE 802.15.4 standard interface. To incorporate this feature, Xbee^®^ RF Modules [21] with antennas were selected from Digi International. There are two types of these modules that can operate in Europe, each using a different frequency: 868 MHz [22] and 2.4 GHz [23]. The weather station was developed to allow the operation of both types, being only necessary to change the configuration. Since the RPI does not have a socket to accommodate the Xbee, a printed circuit board (PCB) was designed for that purpose. Besides the socket, the PCB includes the ADC and connections to plug the power, sensors and buttons. The developed board not only provides a place holder for the Xbee and enables the communication, but it is also ready to control its sleep state. This is particularly useful since this is a self-powered device and awaken 2.4 GHz or 868 MHz Xbee modules have a power consumption of about 37 and 60 mA, respectively. These values drop to around 0.05 mA in sleep mode, justifying the use of sleep control. This board acts as a bridge between the external instrumentation and the RPI. It connects to the RPI via a ribbon cable.

Besides the power saving options adopted, and to accomplish the self-powering goal, a solar panel and a battery were incorporated. To determine their energy capacity, it was assumed that the battery should be able to power the full system for at least 24 h, without any recharge, and the solar panel should be able to charge during the day the energy consumed overnight. From data collected through experiences and by considering safety margins, the requirements for the battery and the solar panel were determined as 10 Ah [24] and 12 V, 1.2 A [25], respectively.

To increase the device sustainability and versatility, a charger prioritizing circuit was included. The objective of this implementation is to allow the user to connect the unit to the power grid, as backup. The prioritizing circuit is a micro-controller that is able to select between three power sources according to a priority list. The circuit has three inputs named V1, V2 and V3, being V1 the one with highest priority and V3 the one having least priority. At each instant, the selected power source is the one with highest priority that is valid according to its voltage. Valid voltages are configured during circuit development by recurring to the adjustment of voltage dividers. In the weather station circuit, the battery is connected to V1, meaning that only when the battery voltage drops below its cutoff voltage, V2 will be activated. When V2, the power grid, is activated, it will power the unit until the battery charge goes over a prescribed threshold, making V1 to return to its valid state. The third power input, V3, is not being used. The circuit selected to implement this behavior was the LTC4417 from Linear Technology [26].

## 3. Software

In the previous section, all core and peripheral hardware components selected for the development of the weather station were enumerated and described. To bring them all together and to create a weather station the software are key parts.

**Figure 2:**
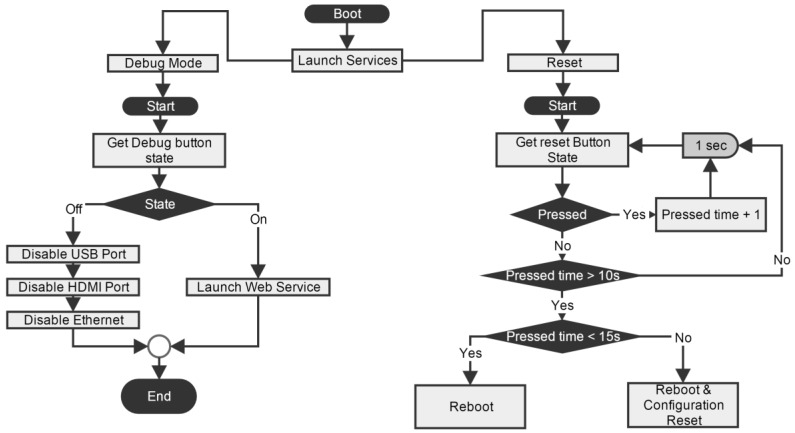
Signal flow diagram of the reset and the power saving buttons monitor services.

As mentioned, a toggle button is used to select the mode of operation and a push button to restart the RPI. Associated with the toggle button there is a service that executes when the RPI is initiated. The service checks the state of the button and performs its actions accordingly. If the device is in debug mode no services are disabled and a web service is started. On the other hand if the device is in power saving mode the web server is not initiated and services related to the Ethernet, HDMI and USB bus are disabled. The state of this button is only verified when the device is initiated. The reset button is checked at every second by a service developed for that purpose. If the button is pressed between 10 and 15 s, the RPI restarts; if it is pressed longer, besides restarting it also resets the network configurations and the administrator password that gives access to the web page. A graphical overview of these services is shown in Figure 2.

The web page is accessible by Ethernet when the RPI is running on Debug mode and, therefore, initializes the web server. The web page’s purpose is to allow changes in configurations, definitions and data visualization (measurements and predictions). Regarding the configuration section, it is possible to change the sampling interval, the prediction interval and some Xbee definitions. The sampling interval can be set from a minimum of 30 s to a maximum of 5 min. The prediction interval must be defined as a multiple of the sampling interval. It can only be set during the initial configuration of the weather station. The webpage also enables the user to change configurations in the Xbee module, such as destination addresses, sleep modes, encryption options, antenna type and additional configurations.

Although the mentioned services are important for the unit to accomplish its objectives, the most important part of the weather station software is the data acquisition part. When the station is turned on, a service is initialized that reads the sensor values at the user-defined rate and transmits the data through the 802.15.4 wireless interface. The values are stored in the RPI using a Sqlite database and marked as sent if the destination device receives the values. Otherwise, if they are not received, they are marked as not sent and scheduled for retransmission later. Sqlite was chosen due to its simplicity and low processing requirements, as it does not possess a database engine and is simply based on files and an interface library. The database is composed of three tables: Config, measurement and predicted values. The config table stores configuration parameters, the measurement table stores the data acquired from the sensors and the predicted table stores the predictions.

The predictions are computed by a program that is executed at every prediction interval. The program is initiated by the cron service, a task scheduling service that exists in most Linux distributions. The predictions are computed by one of two algorithms: A Nearest Neighbors (NEN) algorithm or an Artificial Neural Networks (ANN) based algorithm.

During the first day of deployment, the weather station does not provide any predictions as it does not have the necessary historical data. After acquiring a full day of data, it starts using the NEN algorithm to calculate the predictions of the relative air humidity, air temperature and global solar radiation. It keeps using this methodology until it receives an ANN parameters file through the 802.15.4 module. The parameters file is sent to the weather station by the receiver device, when enough data has been acquired to enable a successful design of the three ANNs. When the program detects the existence of a parameters file, it starts using the ANNs approach to make the predictions. The values acquired are maintained in the RPI for the number of days required to compute the predictions. This number, which is configurable through the webpage, depends on the maximum delay that is used at the inputs of the forecasting algorithms.

## 4. Prototype

To complete the unit, it was necessary to search and select a few more components to allow its outdoor deployment. To hold and protect most of the electronic components, an IP 65 compliant box was used. According to the International Protection Rating, an IP 65 [27] box provides enough protection for an outdoor environment, since it offers a full protection against dust and water jets. The box encloses all the electronic devices, except the sensors that must be located outside the box. The radiation sensor does not require any special protection since it was developed to be directly exposed to the environment. On the contrary, the temperature and humidity sensor is protected by a solar radiation shield, which protects the sensor while maintaining the relevant characteristics of the atmosphere. The prototype also has a mounting point to allow attaching it to a 2 m mast.

**Figure 3:**
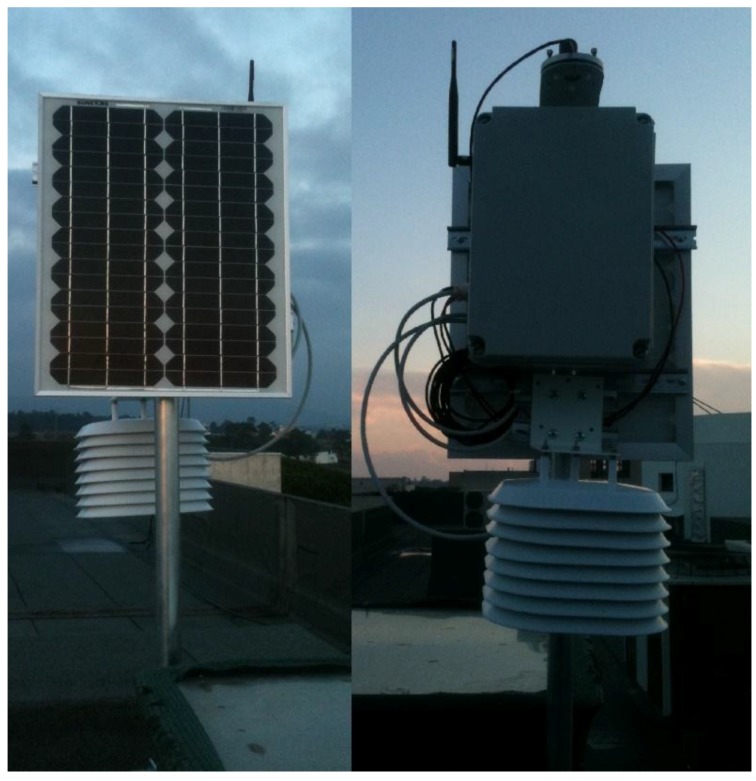
Two sides of the weather station prototype.

In Figure 3, it is possible to see the prototype of the meteorological station fully built and deployed. The station was deployed on the terrace of one of the University buildings, close to an already working data-logger based weather station. The site of deployment was chosen to allow comparison of the acquired data from both stations.

## 5. Predictive Models

Two predictive models are used to generate forecasts of the three measured atmospheric variables: A NEN algorithm and an ANN-based approach. Both compute one-step-ahead predictions, and are iterated in a multi-step fashion to generate longer forecasts up to a given prediction horizon. These techniques are briefly described in this Section; further details can be found in the references provided.

The NEN (for details, please see [28]) uses pattern matching to compute the predictions. Two parameters, *d* and *n*, are necessary: *d* corresponds to the number of full days used to search for the best matching patterns (neighbors); and *n* is the number of *closest* (in the Euclidean sense) neighbors that will be averaged to compute the one-step-ahead prediction.

**Figure 4:**
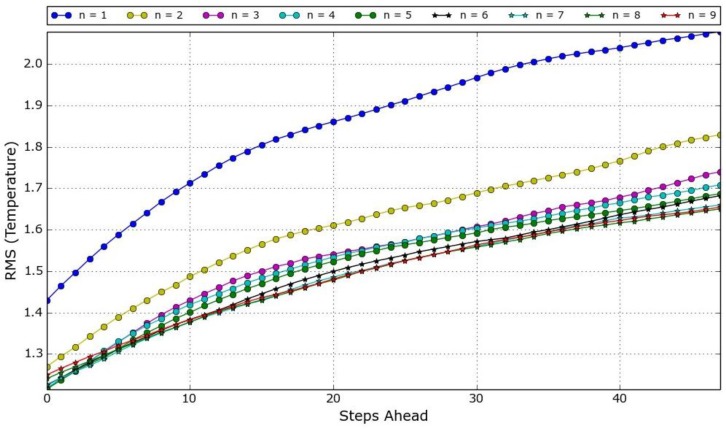
Evolution of the RMSE of the air temperature over the prediction horizon using *d* = 35 for all *n* values.

Using two months of existing data (from 1 July 2014, until 31 August 2014), a number of experiments were executed in order to determine the best (*n*, *d*) pair for each measured variable. For that, *d* and *n* were varied, and for each combination the Root Mean Square Error (RMSE) over the instants of the prediction horizon was computed. The search space consisted of *d* = {7, 14, 21, 28, 35} and *n* = {1, 2, …, 8, 9}. From the experiments, it was concluded that for air relative humidity the pair (21, 4) should be used, (35, 4) for air temperature, and (14, 3) for global solar radiation. An illustration for the air temperature model, using 35 days of data and all values of *n*, is presented in Figure 4. As it can be seen, at the end of the prediction horizon the difference between *n* = 3 and *n* = 9 is about 0.1 °C. Although the pair (35, 4) is not the one that produces the lowest RMSE, it achieves good performance on the first steps, an acceptable performance at the final steps, and allows fewer computations to generate the prediction, therefore also contributing to save some energy.

The ANN-based approach uses, for model design, an existing Multi-Objective Genetic Algorithm (MOGA) framework. This approach can be included in a large set of design algorithms that combine different evolutionary algorithms with derivative-based ones, which can be seen, for instance, in [29,30,31]. Details on the use of the MOGA for model design can be sought, for instance, in [32], therefore it will be briefly described here.

The MOGA is used to design Radial Basis Function (RBF) Neural Network (NN) models, by selecting combinations of input variables (and their lags), as well as the number of neurons, that optimize pre-specified model performance criteria.

The output of a RBF model is given by:

(1) $$o\left[k\right]=w_{l+1}+\sum_{j}^{l}w_{j}e^{-\frac{{\left\|i\left[k\right]-C(j)\right\|}_{2}^{2}}{2\sigma_{j}^{2}}}$$

In Equation (1), *o*[*k*] denotes the output, at instant *k*, *i_j_*[*k*] is the *j*th input at that instant, **w** represents the vector of the linear weights, **C**(*j*) represents the vector (extracted from the **C** matrix) of the centers associated with the hidden neuron *j*, *σ_j_* is its spread, and ‖ ‖_2_ represents the Euclidean distance.

As dynamic models are considered, the RBFs are used as Nonlinear Auto-Regressive (NAR) models. Denoting as *y* the modelled variable, the estimation ($\hat{y}$), at instant *k*, can be given as:

(2) $$\hat{y}\left[k\right]=f\left(y\left[k-d_{o_{1}}\right],\ldots ,y\left[k-d_{o_{n}}\right]\right)$$

In Equation (2), *f* represents the RBF model Equation (1), which means that its argument (the delays of *y*) represent the network input, **i**[*k*], being $d_{o_{i}}>0$. As the objective is to determine the evolution of the variables over a prediction horizon, Equation (2) is iterated over that horizon. For *k* +1, we shall have:

(3) $$\hat{y}\left[k+1\right]=f\left(y\left[k+1-d_{o_{1}}\right],\ldots ,y\left[k+1-d_{o_{n}}\right]\right)$$

Depending on the indices of the delays, for the steps within the prediction horizon, we may not have measured values for one or more terms in the argument of Equation (3). These must be obtained using previous predictions. This way, the computation of the forecast over a given prediction horizon is an iterative process.

The design of models by the MOGA framework involves the determination of:

- The inputs: In this case, this is translated in the number of delays to use, and their values;
- The number of hidden neurons (*l* in Equation (1));
- The model parameters: **C**, **w** and **σ**.

**Figure 5:**
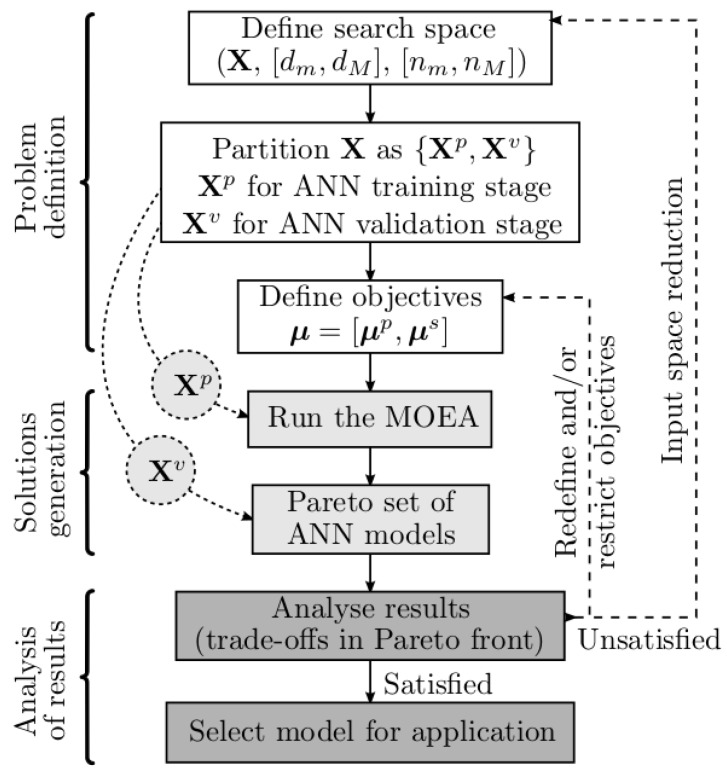
MOGA Worflow (reprinted from [32]).

Before being evaluated in MOGA, each model has its parameters determined by a Levenberg-Marquardt algorithm [33,34] minimizing an error criterion that exploits the linear-nonlinear relationship of the RBF NN model parameters [35,36]. The initial values of the nonlinear parameters (C and **σ**) are chosen randomly, or with the use of a clustering algorithm, **w** is determined as a linear least-squares solution, and the procedure is terminated using the early-stopping approach [37] within a maximum number of iterations. The workflow of MOGA can be seen in Figure 5.

MOGA requires that the data used to develop the models is divided into three data sets: a training set to estimate the model parameters, a testing set for terminating the training, and a validation set to compare the performance of the models obtained by the MOGA (as the MOGA uses a multi-objective formulation, its results are not a single solution, but a set of non-dominated solutions). This data division is automatically executed by an algorithm, *AppoxHull*, which also ensures that the vertices in the approximate convex hull of the whole data are included in the training set. For more details on *ApproxHull*, please see [38].

The design of NNs by a MOGA is a time-consuming process. For this reason, it is executed in a cluster of processors. It is also an iterative process, which means that the results obtained in one design experiment enable the user to fine tune the parameters of a next experiment.

As described before, the first predictions are obtained using the NEN algorithm, after storing one day of data, *i.e.*, (*n*, *d*) = (1, 1). These values will be increased every day, until the desired values for each model can be achieved. This way, all the processing is done at the weather station.

The usage of the ANN models requires their design, which is done externally. The process of model design starts when one of two conditions is met: after a fresh start, when enough data is collected; or when a degradation of the forecasts is identified. The process is implemented by an Hyper-Text Transfer Protocol (HTTP) based Web Service (WS) using a basic web server authentication. It accepts a POST of data that is triggered by the Weather Station Receptor (WSR) device or by the weather station, when the conditions mentioned above are detected. After the POST of data, a simple protocol ensures that the weather station will receive the expected ANNs parameters.

When the WS receives a POST of data, it manages the authorization, the ANN design tasks, and offloads the ANN design work to another system. Additionally, an email notification of this event is sent to the technical team. For each weather station, only one design task is possible at any time.

**Figure 6:**
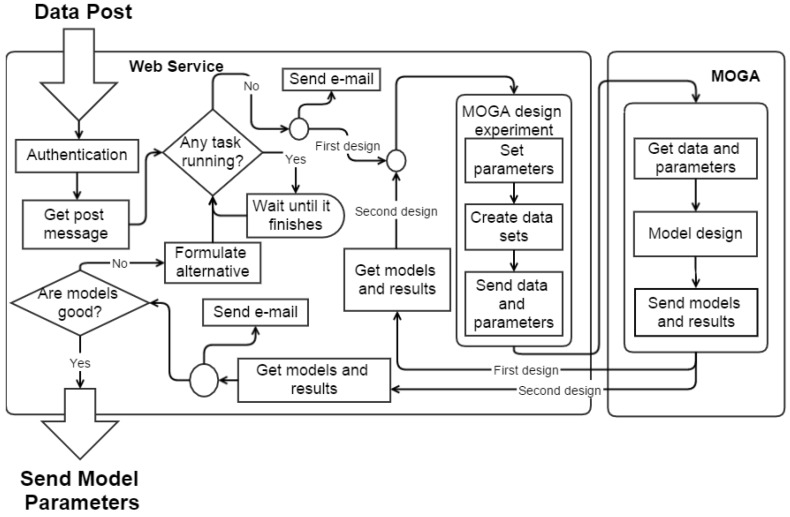
WS signal flow diagram.

When a new task is created in the WS, it spawns two MOGA design experiments for each variable. The first uses the parameters detailed above, while in the second, some MOGA parameters are fine tuned, depending on the results of the first execution. In this case, the RMSEs for the training and testing error are set as restrictions, as well as the model complexity. The number of neurons, the number of inputs and the set of lags considered are also decreased, therefore reducing the search space of MOGA. When the second design is finished, the model parameters are sent back to the WS that notifies the technical team of the design conclusion. If the new ANN parameters are considered good, the process is validated and the WS will send them to the weather station. If the validation is faulty, the technical team must analyze the problem, formulate alternative MOGA experiments and afterwards submit validated parameters to the WSR. A signal flow diagram of the WS is presented in Figure 6.

## 6. Results

The intelligent weather station was deployed in the terrace of one of the buildings of the Gambelas campus of the University of Algarve, Faro, Portugal, between 22 February 2015, 03:00:00 + 00:00 and 7 April 2015, 14:35:00 + 01:00. During this period, 12,800 samples of the three atmospheric variables were collected. During the first 35 days, the forecasts within a prediction horizon of 48 steps ahead (4 h, as the prediction interval considered was 5 min), were obtained by the NEN algorithm. After that, and with 10,000 values acquired for each variable, the neural network models were designed and transmitted to the weather station by the WS. The results of the forecasts for the three variables obtained by the RBF models, for the last 1350 samples (approximately the last five days) were compared with the results obtained by the NEN, with the parameterizations already mentioned, as well as (*n*, *d*) = (2, 2) and (7, 4).

The RBF models were designed with two iterations of MOGA. In the MOGA experiments executed for this work, the parameters employed in the first iteration were: number of neurons in the hidden layer limited between two and 30; number of input terms for each neuron between one and 15; number of RBF NN training trials 10; maximum number of training iterations 50; population size 100. The selected model design objectives are: the RMSE obtained in the training and test data sets, and the model complexity. In the second iteration, the MOGA parameters have been restricted based on the results obtained on the non-dominated set. The previous objectives were set as restrictions, with goals determined as the average of the previous results in the non-dominated set, and a further objective, the RMSE over the prediction horizon, was incorporated.

### 6.1. Atmospheric Temperature

*ApproxHull* found 696 vertices in the data convex hull. The number of samples for the training, testing and validation data sets were 2888, 962 and 964, respectively. The MOGA yielded 83 non-dominated solutions. From these, a six-input model Equation (4) was selected:

(4) y[k]=f(y[k−1],y[k−2],y[k−6],y[k−11],y[k−277],y[k−299])

**Figure 7:**
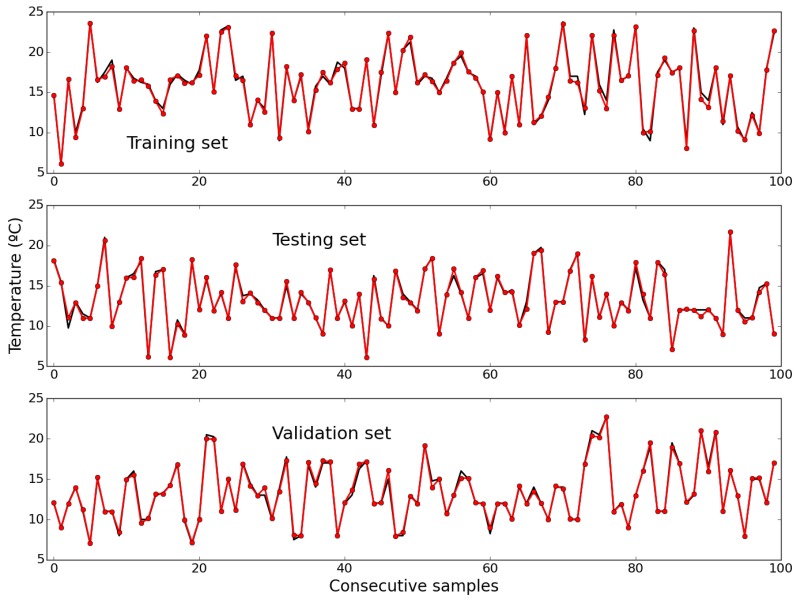
Measured (black) and estimated (red) values of Temperature, for the first 100 samples of the training (**Top**); testing (**Middle**); and validation (**Bottom**) data sets.

Figure 7 shows the measured values (in black) and the values estimated by the model (in red), for the first 100 samples of the training, testing and validation sets, respectively. The RMSE values obtained with these data sets were 0.36, 0.31 and 0.32, respectively. It may be observed that an almost exact fitting has been achieved in all data sets, which is an excellent sign about the generalization capability of the model.

**Figure 8:**
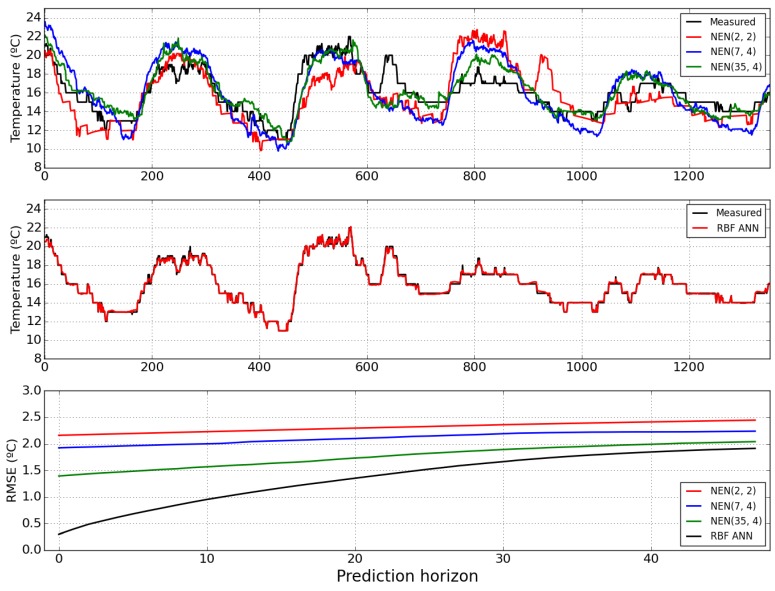
One-step-ahead error for the NEN algorithm (**Top**) and the RBF ANN (**Middle**); considering the last 1350 data points. Evolution of the RMSE (**Bottom**), along the prediction horizon instants, for air temperature forecasts obtained by the RBF model (black), and the NEN algorithm: Red (2, 2), blue (7, 4) and green (35, 4).

Figure 8 shows the air temperature one-step-ahead predictions obtained by the NEN algorithm and the RBF ANN for the last 1350 samples, as well as the evolution of the RMSE along the prediction horizon. By comparing the top and middle plots, it is clear that the best performance is obtained by the RBF ANN model. Regarding the prediction error over the instants of the prediction horizon, the bottom plot shows that the RBF ANN, as expected, achieves also the best results. It may also be concluded that the NEN method performance is positively influenced by parameterizations that use more data. The NEN (35, 4) is comparable to the RBF in the end part of the horizon, but much worse over the first half.

Table 1 summarizes the one-step-ahead RMSE and presents the sum of the RMSE over the prediction horizon instants, for the four models. It is evident, among the NEN parameterizations, that the best results are obtained by the selected one, (35, 4), and that the best performance overall is achieved by the ANN model.

**Table 1:** Root mean square errors (RMSEs) of Temperature.

Model	*RMSE*_1_	$\sum_{i=1}^{48}{RMSE}_{i}$
NEN (2, 2)	2.17	111.06
NEN (7, 4)	1.93	101.52
NEN (35, 4)	1.39	84.79
RBF	0.30	65.46

### 6.2. Atmospheric Relative Humidity

*AppoxHull* found 1169 vertices in the data convex hull. The number of samples for the training, testing and validation was 2888, 962 and 964, respectively. The MOGA yielded 83 non-dominated solutions. From these, a six-input model Equation (5) was selected:

(5) y[k]=f(y[k−1],y[k−2],y[k−7],y[k−8],y[k−12],y[k−17])

**Figure 9:**
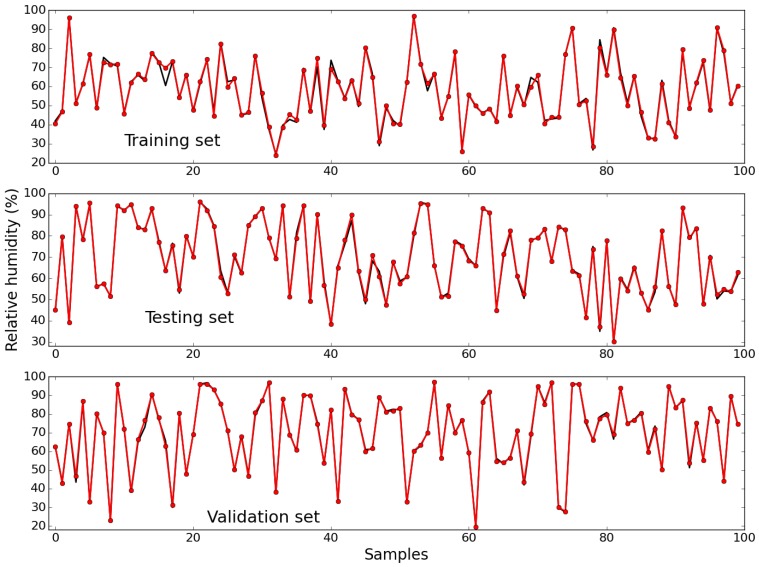
Measured (black) and estimated (red) values of relative humidity, for the first 100 samples of the training (**Top**); testing (**Middle**); and validation (**Bottom**) data sets.

Figure 9 depicts the measured values (in black) and the values predicted by the model, for the first 100 samples of the training, testing and validation sets, respectively. The corresponding RMSE values obtained with these data sets were 1.46, 1.03 and 1.07, respectively. As for air temperature, an almost perfect fitting was obtained in all cases.

**Figure 10:**
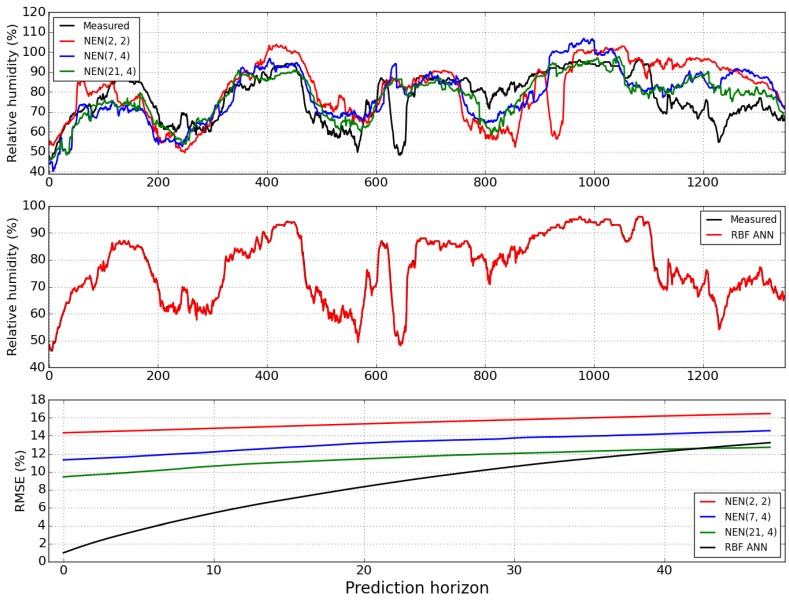
One-step-ahead error for the NEN algorithm (**Top**) and the RBF ANN (**Middle**), considering the last 1350 data points. Evolution of the RMSE (**Bottom**), along the prediction horizon instants, for air relative humidity forecasts obtained by the RBF model (black), and the NEN algorithm: Red (2, 2), blue (7, 4) and green (21, 4).

Figure 10 illustrates the one-step-ahead predictions obtained by the NEN algorithm with the three parameterizations and the RBF ANN, for the last 1350 samples, as well as the evolution of the RMSE along the prediction horizon for the four models. Please notice that, for relative humidity, the chosen NEN parameterization was (21, 4).

It may be seen that the NEN performance suffers, because relative humidity day-to-day patterns exhibit a stronger variability than air temperature. Figure 10 shows the corresponding result obtained by executing the RBF model (in red). The conclusions that were drawn for air temperature apply also to relative humidity: The best performance was obtained by the RBF NN model, and the parameterization (21, 4) achieved the best NEN results, which are better than those obtained by the RBF at the last few instants of the prediction horizon.

Table 2 presents the summary of results for the relative humidity, considering the four models. As found for the Temperature, among the NEN parameterizations, the best results are obtained by the selected one, and the best performance overall is obtained by the ANN model.

**Table 2:** RMSEs of Relative Humidity.

Model	*RMSE*_1_	$\sum_{i=1}^{48}{RMSE}_{i}$
NEN (2, 2)	14.34	742.17
NEN (7, 4)	11.32	632.72
NEN (21, 4)	8.52	497.36
RBF	0.99	409.43

### 6.3. Solar Radiation

*ApproxHull* found 659 vertices. The number of samples for the training, testing and validation was 2895, 965 and 966, respectively. The MOGA yielded 168 non-dominated solutions. From these, an 11-input model Equation (6) was selected:

(6) $$y\left[k\right]=f\left(\begin{array}{l} y\left[k-1\right],y\left[k-2\right],y\left[k-3\right],y\left[k-5\right],y\left[k-8\right],y\left[k-34\right],\\ y\left[k-35\right],y\left[k-37\right],y\left[k-281\right],y\left[k-289\right],y\left[k-290\right] \end{array}\right)$$

**Figure 11:**
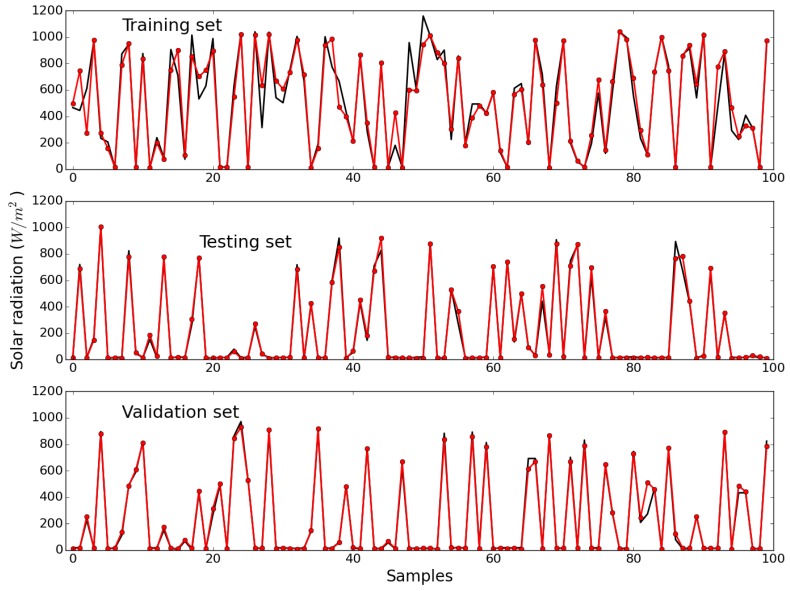
Measured (**black**) and estimated (**red**) values of solar radiation, for the first 100 samples of the training (**Top**); testing (**Middle**); and validation (**Bottom**) data sets.

Figure 11 shows the measured values (in black) and the values estimated by the model, for the first 100 samples of the training, testing and validation sets, respectively. The RMSEs obtained with these data sets were 71.35, 31.2 and 38.18, respectively. Although the fittings are excellent, as for the other variables, regarding solar radiation the error in the training set is, in comparison, higher. This was a consequence of an increase of cloudiness during those days, which resulted in increased uncertainty and consequent degradation of predictive performance. Please note that the RMSE in the training set is expected to be higher than in the other sets, as the training set includes the convex hull samples.

**Figure 12:**
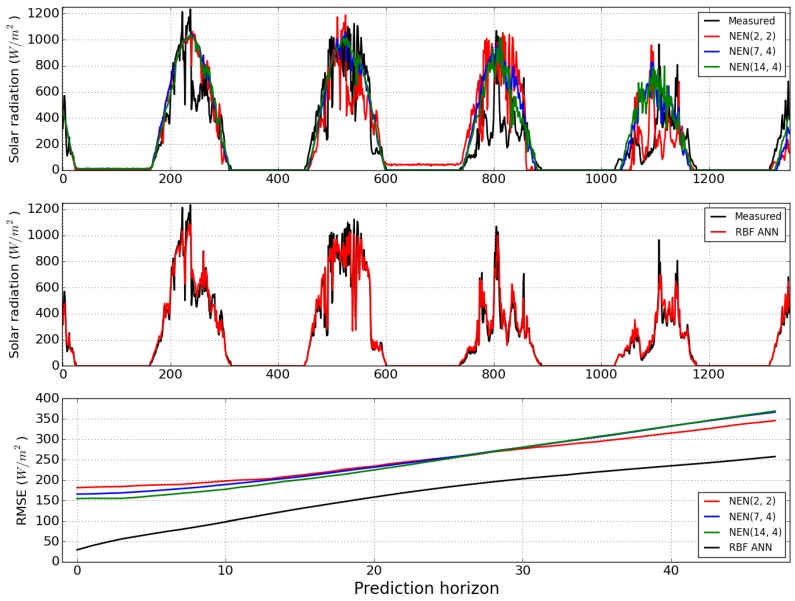
One-step-ahead error for the NEN algorithm (**Top**) and the RBF ANN (**Middle**), considering the last 1350 data points. Evolution of the RMSE (**Bottom**), along the prediction horizon instants, for solar radiation forecasts obtained by the RBF model (black), and the NEN algorithm: Red (2, 2), blue (7, 4) and green (14, 4).

Figure 12 shows the one-step-ahead predictions obtained by the NEN algorithm with the three parameterizations, and by the RBF ANN model, for the last 1350 samples, as well as the evolution of the RMSE over the prediction horizon instants for all the models. Please notice that, for this variable, the chosen parameterization was (14, 4). By comparing the top and middle plots it may be concluded that the RBF ANN achieves the best results. This may be confirmed by looking at the bottom plot, which illustrates the evolution of the RMSE, along the prediction horizon instants, obtained by the four models considered. Contrary to the previous two weather variables, for solar radiation none of the NEN models achieves a comparable performance with the RBF ANN in any part of the prediction horizon.

Table 3 shows the one-step-ahead RMSE as well as the sum of the RMSE over the prediction horizon instants, for all the models. As found for the two other variables, among the NEN parameterizations, the best results are obtained by the selected one, and the best performance overall is achieved by the ANN model.

**Table 3:** RMSEs of Solar Radiation.

Model	*RMSE*_1_	$\sum_{i=1}^{48}{RMSE}_{i}$
NEN (2, 2)	132.22	12,109
NEN (7, 4)	122.26	12,173
NEN (14, 4)	154.67	11,951
RBF	29.49	7850

## 7. Conclusions

In this paper, we have shown the hardware and software design of an intelligent weather station which, besides offering wireless communication and energy autonomy, provides not only measurements of atmospheric variables, but also their forecasts, over a prediction horizon.

Two types of forecasting methods are available in the weather station: a Nearest Neighbor algorithm, which is completely local to the station, and another based on Artificial Neural Network models, which, for their design, requires an auxiliary computer system to perform optimized model design. It was shown that the forecasts obtained by the ANN models are substantially better than the ones given by the NEN algorithm.

An accurate comparison of the prediction results with other approaches is difficult because the data, as well as the prediction interval, are not the same and, typically, a prediction horizon of just one step is considered in these approaches. In order to have a general idea of the quality of the RBF forecasts of the intelligent weather station, compared with other solutions, it must first be assumed that similar predictions will occur when the prediction interval is changed. Assuming this, it can be pointed out that, in [5], different 1-step forecasting methods of solar radiation were compared, considering a prediction interval of 1 h. Their RMSEs lied between a range of [48.8 59.5] W/m^2^. In our case, the RMSE is 29.5 W/m^2^ for a prediction interval of 5 min. In [4], considering also a prediction interval of 1 h, the mean absolute percentage error (MAPE) obtained for the solar radiation of one cloudy day is 9.65%, while this index, computed for the RBF results using the data used in Section 6.3, is 7.78%. In [3], one-step-ahead forecasts of air temperature and relative humidity are presented. Their RMSEs lie in the range of [1.58 1.63] °C and [4.25 4.47]%, while in the ANN approach presented in this paper these values are 0.3 °C and 0.99%.

If any of the approaches referenced above would be applied for a specific site, a weather station would have to be installed in that location, models would have to be designed externally, using data that should be transmitted in real-time from that weather station, and one-step-ahead predictions would have to be computed, in real-time, externally. The intelligent weather station proposed in this work is a complete and autonomous solution to this problem, besides enabling to have excellent quality predictions over a user-defined prediction horizon, and not for a single step. As such, we anticipate that a commercial version of such a device will have a large range of practical applications, examples being in PV plants, energy building management systems as well as in agricultural applications.
